# The use of the Hungarian Test Your Memory (TYM-HUN), MMSE, and ADAS-Cog tests for patients with mild cognitive impairment (MCI) in a Hungarian population: a cross-sectional study

**DOI:** 10.1186/s12888-020-02982-6

**Published:** 2020-12-01

**Authors:** Szabolcs Garbóczy, Éva Magócs, Gergő József Szőllősi, Szilvia Harsányi, Anikó Égerházi, László Róbert Kolozsvári

**Affiliations:** 1 Gyula Kenézy University Hospital, Department of Adult Psychiatry, University of Debrecen, Debrecen, Hungary; 2 Department of Psychiatry, Clinical Center, University of Debrecen, Debrecen, Hungary; 3 Faculty of Public Health, University of Debrecen, Debrecen, Hungary; 4 Department of Family and Occupational Medicine, Faculty of Public Health, University of Debrecen, Postal Adress: Móricz Zs. krt. 22, Debrecen, 4032 Hungary

**Keywords:** Dementia, Alzheimer’s disease, Mild cognitive impairment, Diagnosis, Test, Early detection

## Abstract

**Background:**

Mild cognitive impairment (MCI) often presages the development of Alzheimer’s disease (AD). Accurate and early identification of cognitive impairment will substantially reduce the burden on the family and alleviate the costs for the whole society. There is a need for testing methods that are easy to perform even in a general practitioner’s office, inexpensive and non-invasive, which could help the early recognition of mental decline. We have selected the Test Your Memory (TYM), which has proven to be reliable for detecting AD and MCI in several countries. Our study was designed to test the usability of the Hungarian version of the TYM (TYM-HUN) comparing with the Mini-Mental State Examination (MMSE) and the Alzheimer’s Disease Assessment Scale-Cognitive Subscale (ADAS-Cog) in MCI recognition in the Hungarian population.

**Methods:**

TYM test was translated and validated into Hungarian (TYM-HUN) in a previous study. The TYM-HUN test was used in conjunction with and compared with the MMSE and the ADAS-Cog. For our study, 50 subjects were selected: 25 MCI patients and 25 healthy controls (HC). Spearman’s rank correlation was used to analyse the correlation between the scores of MMSE and ADAS-Cog with TYM-HUN and the receiver operating characteristic (ROC) curve was established.

**Results:**

MCI can be distinguished from normal aging using TYM-HUN. We established a ‘cut-off’ point of TYM-HUN (44/45points) where optimal sensitivity (80%) and specificity (96%) values were obtained to screen MCI. The total TYM-HUN scores significantly correlated with the MMSE scores (*ρ* = 0.626; *p* < 0.001) and ADAS-Cog scores (*ρ* = − 0.723; *p* < 0.001).

**Conclusions:**

Our results showed that the TYM-HUN is a reliable, fast, self-administered questionnaire with the right low threshold regarding MCI and can be used for the early diagnosis of cognitive impairment.

## Background

Having regard to the global burden of dementia, comprehensive research on possible instruments to properly determine cognitive decline has received a great amount of interest.

### Diagnostic criteria

The mild cognitive impairment (MCI), which can be considered as a prodrome of dementia, has no universal diagnostic criteria but the following considerations are generally accepted: memory complaint; memory impairment for age and education; preserved general cognitive functions; intact activities of daily living. In contrast, in dementia everyday functionality is no longer maintained and significant cognitive impairment occurs in one or more areas [1–5].

### Epidemiology

Dementia is a disease that afflicts a large proportion of the elderly. The WHO estimates that it affects around 50 million people worldwide and that there are around 10 million new cases a year [6].

The process of developing dementia may remain clinically unnoticed for a long time: the onset of molecular pathology can precede the onset of symptoms by up to 20 years. To intervene effectively and promptly, we need to recognize dementia before it fully develops clinically [7].

In light of this, it is increasingly crucial to have appropriate screening instruments available that are sufficiently accurate and have convenient merits in the early identification of cognitive deficit. These should preferably be screening tests that have outstanding sensitivity and specificity in the identification of cognitive impairment at the early stage including MCI and mild dementia.

### Assessment

The most commonly used procedures in Hungary for the identification of AD are the Mini-Mental State Exam (MMSE) and the Early Mental Test developed by the University of Szeged [8, 9]. There is no international consensus on which is the best test and with which cut-off points. In recent years, the Montreal Cognitive Assessment (MoCA) [10] and the Short Test of Mental Status [11] have been used for this purpose, the Hungarian version of which is not yet available [12].

The MMSE has been shown to be particularly useful in identifying dementia with a short filling time (around 5–10 min). In a systematic review, the authors found that the sensitivity and specificity of MMSE were 0.81 and 0.89 respectively [13]. Janka et al. translated and validated it into Hungarian, the cut-off point they established was 24 [14]. However, it is unreliable in the early detection and not suitable for distinguishing healthy people from those with MCI [15].

For this purpose, Alzheimer’s Disease Assessment Scale-Cognitive Subscale (ADAS-Cog) [16], which has more reliable properties but is more time-consuming (about 35 to 45 min depending on the number and the extent of cognitive areas affected) is used [17]. Although the potential for improvement of the test is raised by a large literature review, it is acknowledged that the ADAS-Cog is a useful measurement instrument in pre-dementia syndromes [18]. In its Hungarian validated form, outstandingly high values were found in the separation of healthy people from AD patients [19]. Both the sensitivity and the specificity are quite high at the mathematically optimal decision point of 12.15, respectively 96.97 and 91.49%.

We have selected a short, self-administered test developed by the English author Jeremy Brown, which is validated in different countries worldwide, also in Hungary (TYM-HUN) [20, 21]. This is the Test Your Memory (TYM), which examines a fairly wide range of cognitive functions (orientation, attention, executive functions, language, memory, and visuospatial skills) in a short period of time (5–10 min) and provides reliable information to the clinician when properly evaluated [22]. The TYM-HUN test, which was previously translated into Hungarian and validated, was found to be reliably used to screen for dementia using a 35/36 cut-off, with which both the sensitivity and specificity of the test were found to be 94% [21].

The goal of this study was to compare the diagnostic utility of the TYM-HUN with the MMSE and the ADAS-Cog tests for MCI in a Hungarian population. We determined a ‘cut-off’ point of TYM-HUN where optimal sensitivity and specificity values were obtained to screen MCI.

## Methods

### Inclusion and exclusion criteria

Fifty persons, 25 MCI patients and 25 healthy controls (HC), were recruited into the observational cross-sectional study at the Psychiatric Clinic and Hospital of the University of Debrecen between January 2018 and August 2019. Adults (patients/HCs) from the age of 18 and over were included. The diagnosis of MCI was made according to the DSM-5 diagnostic criteria for mild neurocognitive disorders [5]. Patients were seen and diagnosed by a consultant psychiatrist and underwent neurological assessment, MMSE, ADAS-Cog, structural imaging [e.g. computed tomography (CT), magnetic resonance imaging (MRI)], and blood tests. According to the structural imaging results and the laboratory tests, there was no evidence of neurovascular disease or other neurological or systemic diseases. To avoid possible bias due to mood depression, subjects for whom the Beck Depression Inventory (BDI) indicated the presence of depressive symptoms were excluded from further investigation.

HCs were recruited from relatives accompanying patients to the clinic and the hospital, as well as from relatives of patients attending psychiatric outpatients’ departments at these two institutions. The people with a history of neurological disease, memory problems, or brain injury were excluded from the HC group. HCs were included based on the absence of the symptoms, that met the diagnostic criteria for mild neurocognitive disorder (DSM-5) [5]. All participants gave written informed consent and they filled out all our questionnaires completely.

TYM test was translated into Hungarian language and validated for AD (TYM-HUN) [21]. The MMSE, ADAS-Cog, and TYM-HUN tests, and to exclude the depression, the BDI was filled out by the patients and controls, as well.

The age distribution for normality of the sample was tested with the Shapiro-Wilk test and the age difference between the MCI patients and controls was tested with the Mann-Whitney-Wilcoxon rank sum test. We used Fisher’s exact test to investigate the gender distribution and the Kruskal-Wallis test for testing the educational differences.

Spearman’s rank correlation was used to investigate the correlations between the different tests. The receiver operating characteristic (ROC) curve, the sensitivity, specificity, positive (PPV) and negative predictive values (NPV), Youden Index, and area under the curve (AUC) were also established.

We performed the statistical analyses with STATA 11.1 software (Statacorp LP. College Station, TX, USA).

## Results

**Fig. 1:**
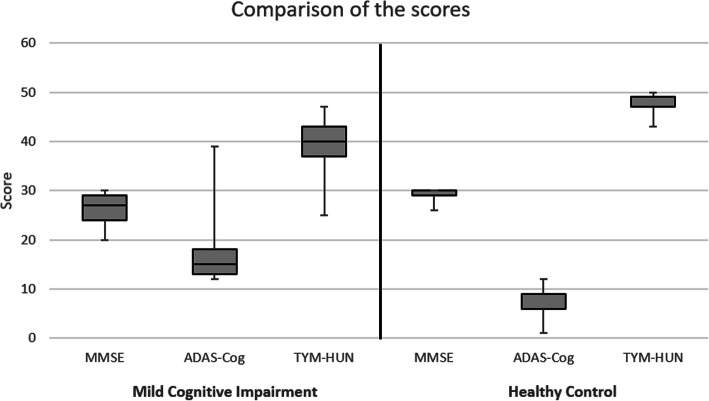
Comparison of the MMSE, ADAS-Cog, and TYM-HUN median scores and interquartile ranges (IQR) between the MCI and HC group with box plots

We evaluated the TYM-HUN results of 50 adults aged 55–84 years. Twenty-five MCI patients (mean age 74.84 ± 6.22 years) and 25 HC participants (mean age 71.32 ± 7.69 years) were investigated. The male to female ratios were 6:19 and 8:17 in the MCI and HC groups, respectively. The MCI patients reached an average score of 39.52 ± 5.73/25 on the TYM-HUN, 26.32 ± 2.98/25 points on the MMSE, and 16.80 ± 6.11/25 on the ADAS-Cog tests. The average results of the HC group members were 47.40 ± 1.68/25 with the TYM-HUN, 29.04 ± 1.06/25 with the MMSE, and 5.80 ± 3.64/25 with the ADAS-Cog tests. We also calculated the median scores and the interquartile ranges (IQR) of the tests (MMSE, ADAS-Cog, and TYM-HUN) and these are presented in Fig. 1.

The age distribution of the sample was significantly (*p* < 0.001) different from the normal distribution, and no significant (*p* = 0.057) difference was observed between the MCI and HC group’s mean age. There was no significant difference between the gender distribution of the two groups (*p* = 0.754). These calculations suggest that our sample was comparable with respect to age and gender. In the case of patients with MCI, no significant difference was found between the subjects’ mean TYM-HUN score when comparing them according to their gender (male = 43.33, female = 38.32, *p* = 0.060). There were no significant differences between when the educational level was analysed (primary = 38.00; secondary = 38.60; tertiary = 41.55; *p* = 0.467).

**Fig. 2:**
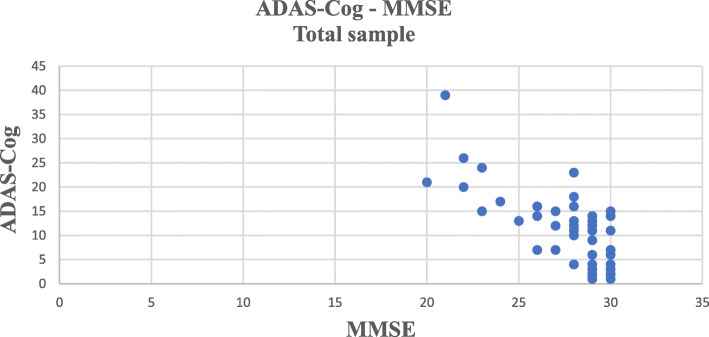
The correlation between ADAS-Cog and MMSE in the total sample (*n* = 50)

Spearman’s rank correlation was used to analyse the correlation between the scores of MMSE and ADAS-Cog with TYM-HUN. The total TYM-HUN scores significantly correlated with the MMSE scores (*ρ* = 0.626; *p* < 0.001) and ADAS-Cog scores (*ρ* = − 0.723; *p* < 0.001). Negative correlation can be seen between ADAS-Cog and MMSE (*ρ* = − 0.67, *p* < 0.001). (Fig. 2).

**Fig. 3:**
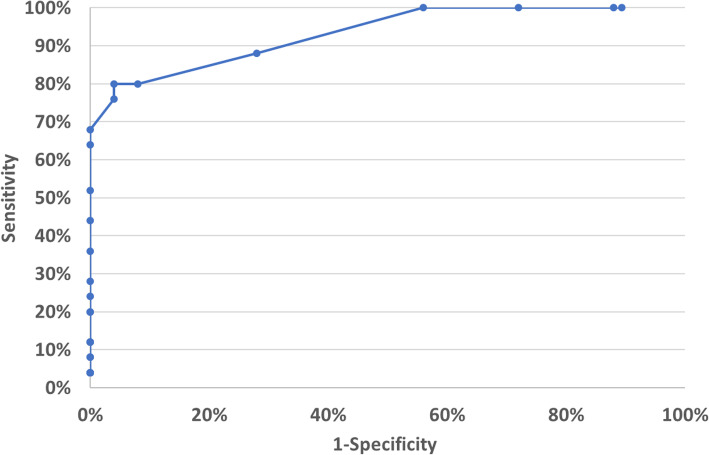
The ROC curve shows the Sensitivity and 1-Specificity of the TYM-HUN test with MCI patients

According to the Youden Index (Y = 76.00%) and the square of the distance (d^2^ = 4.16%), the ideal cut-off score should be at 44/45 between HC and MCI patients (Table 1). With this cut-off point the true positive rate is 80.00%, the true negative rate is 96,00%, the PPV is 95,24% and the NPV is 82,76%. If this cut-off is applied, then according to our sample the area under the ROC curve is 93,20%. The ROC curve, that was created based on the TYM-HUN scores using the presence/absence of MCI as the criterion variable, is presented in Fig. 3.

**Table 1:** The scores of the TYM-HUN, HC and MCI (diagnosed with ADAS-Cog) patients and the sensitivity, specificity, positive (PPV) and negative predictive values (NPV)

TYM-HUN	HC	MCI	Sensitivity	Specificity	PPV	NPV
25	0	1	4%	100%	100%	51%
26	0	0	4%	100%	100%	51%
27	0	0	4%	100%	100%	51%
28	0	0	4%	100%	100%	51%
29	0	1	8%	100%	100%	52%
30	0	0	8%	100%	100%	52%
31	0	1	12%	100%	100%	53%
32	0	0	12%	100%	100%	53%
33	0	0	12%	100%	100%	53%
34	0	2	20%	100%	100%	56%
35	0	0	20%	100%	100%	56%
36	0	1	24%	100%	100%	57%
37	0	1	28%	100%	100%	58%
38	0	2	36%	100%	100%	61%
39	0	2	44%	100%	100%	64%
40	0	2	52%	100%	100%	68%
41	0	3	64%	100%	100%	74%
42	0	1	68%	100%	100%	76%
43	1	2	76%	96%	95%	80%
44	0	1	80%	96%	95%	83%
45	1	0	80%	92%	91%	82%
46	5	2	88%	72%	76%	86%
47	7	3	100%	44%	64%	100%
48	4	0	100%	28%	58%	100%
49	4	0	100%	12%	53%	100%
50	3	0	100%	11%	50%	100%

## Discussion

The increasing number of the patient with dementia and MCI, the financial and social burdens of these diseases made it necessary, to investigate the possibilities of the earliest detection of the pathological cognitive impairment.

Whilst the Hungarian version of the MMSE is a good diagnostic test for AD, it is not sensitive to detect MCI [15]. The ADAS-Cog can be used to distinguish AD and normal aging however it takes a long time to administer (35–45 min) [17].

According to our findings, the TYM-HUN seems to be a useful tool for the early detection of the MCI. The correlations we found are as strong as we expected between the different measuring instruments.

As Julayanon [23] pointed out, MoCA’s superiority over MMSE in its sensitivity probably stems from a deeper, more detailed, more in-depth examination of delayed recall. MoCA and TYM have very similar qualities in the study of this cognitive function, perhaps if possible, TYM is even more sensitive in this area: more words to recall (6 to 5) and the same learning trials (2) and similar time between immediate and delayed recall (5 min). This phenomenon’s validity is corroborated by the observation that this function is impaired in the early stages of the disease. This assumption is supported by numerous prominent authors [7, 17, 24, 25]. In the previous validation, we found that the reliable cut-off point for dementia detection was 35/36 [21]. This correlates roughly with that the memory subtest, the subtest for the most impaired cognitive area in amnestic MCI (aMCI), in the TYM-HUN test counts 9 points and the ideal cut-off we found for MCI detection was 44/45. This link is hard to miss and it can be admitted that the two may have been connected.

There are other instruments applied to screen for dementia or MCI, such as Self-Administered Gerocognitive Examination (SAGE) [26] or Cognitive Assessment Screening Test (CAST) [27]. The SAGE is a brief cognitive assessment instrument developed in 2010 that is also self-administered and takes about 15 min to complete, and the developers, with a relatively low number of cases, found it particularly useful in detecting MCI: sensitivity 79%, specificity 95% [26]. SAGE is mostly used for community screening and is usually not included in the compilation of major reviews of screening tests [28]. CAST is also a self-administered screening test with advantageous properties with reasonably high sensitivity (88–95%) and specificity (88–100%) [29]. It takes about 15 min to complete. Studies with a relatively low number of cases are available, and is primarily not of diagnostic value, but is suitable for raising the possibility of further investigations [30]. According to our results, while the TYM-HUN is also self-administered and has almost the same characteristics regarding sensitivity (80%) and specificity (96%), it examines many aspects of the cognitive functions in a shorter time (5–10 min) so it can be used outside the hospital settings, for example in the primary care or family medicine practices. Moreover, a telephone version of the TYM test has been developed which makes it easier to reach the patients [20].

We compared the results we obtained with other nations’ findings regarding the usability of TYM in recognizing MCI. While MCI subjects had an average score of 45 in the original publication, our study found it to be 39.52 [22]. In studies conducted in Chile and Japan have found that the 44-points cut-off value is appropriate to distinguish between HC and MCI which is similar to our result. Based on these results, the test has 85.7 and 76% sensitivity, 69 and 74% specificity respectively in the above-mentioned studies, while our results show that these values are 80 and 96% [31, 32]. Polish and French investigators did not find the test useful in differentiating between MCI and HC [33, 34].

Japanese researchers in a larger sample, like us, found no significant difference in test scores with regard to gender and education level [32]. According to French data, there is no correlation between gender and years spent in education (*p* = 0.34) and performance on TYM, but this can be observed regarding age (*p* = 0.004) [24]. According to the Chilean study, the performance was influenced by the highest level of education (β coefficient = 0.31, *p* < 0.001), but age (*p* = 0.849) had no relation to performance on the TYM test [31]. The Polish study (which divided the subjects into two groups according to age and set the dividing line at 75 years) found that age (*p* < 0.003) and the number of years of education (*p* < 0.001) influence the score on TYM [33].

## Conclusions

One of the main strengths of our study is that the TYM-HUN test is the first short, self-administered test in Hungarian language, that can detect MCI. The ADAS-Cog is also sensitive enough, but the administration of the ADAS-Cog is time-consuming and it is difficult to use in the everyday outpatient and primary care settings.

The limitation of the study is the relatively low number of cases, but in most of the other studies (especially from small countries, like Hungary) the clinicians could not find a lot of patients with suspected MCI and who fit the inclusion criteria and are willing to take part in the study. The refusal of the patients had been one of the greatest challenges, maybe because they do not have a sense of illness or do not want to know about the MCI or early stage of AD or fearing the stigmatization.

The TYM-MCI is a recently developed test for the distinguishment of aMCI and normal, and because of its novelty, it is not yet widespread. Although this specially developed version of the test is available for the detection of MCI with a sensitivity of 0.79 and specificity of 0.91, according to our study, TYM-HUN alone can detect patients with MCI in a Hungarian sample [35].

Summarizing, the TYM-HUN test is a reliable and easy-to-administer tool for assessing people with suspected MCI in Hungarian language. The adapted versions into different languages of the TYM test can be useful tools to recognize the early stages of dementia in hospital, outpatient, and primary care settings and make an international comparison between the countries’ population regarding the different forms of cognitive impairment, as well.

## Data Availability

The datasets used and analysed during the current study are available from the corresponding author on reasonable request.

## Abbreviations

AD: Alzheimer’s disease
ADAS-Cog: Alzheimer’s Disease Assessment Scale-Cognitive Subscale
aMCI: Amnestic mild cognitive impairment
AUC: Area under the curve
BDI: Beck Depression Inventory
CAST: Cognitive Assessment Screening Test
CT: Computed tomography
DSM-5: Diagnostic and Statistical Manual of Mental Disorders, 5th Edition
HC: Healthy control
IQR: Interquartile ranges
MCI: Mild cognitive impairment
MMSE: Mini-Mental State Examination
MoCA: Montreal Cognitive Assessment
MRI: Magnetic resonance imaging
NPV: Negative predictive value
PPV: Positive predictive value
ROC: Receiver operating characteristic
SAGE: Self-administered Gerocognitive Examination
TYM: Test Your Memory
TYM-HUN: Hungarian version of the Test Your Memory
WHO: World Health Organization
